# Case series: *Stenotrophomonas maltophilia* in pediatric oncology patients

**DOI:** 10.1002/cnr2.1982

**Published:** 2024-02-28

**Authors:** Sanila Sarkar, Lea M. Stitzlein, Emily Rav, Miriam B. Garcia, Shehla Razvi, Michael Chang, Ramia Zakhour

**Affiliations:** ^1^ Division of Pediatrics The University of Texas MD Anderson Cancer Center Houston Texas USA; ^2^ Division of Infectious Diseases McGovern Medical School at UT Health Houston, and Children's Memorial Hermann Hospital Houston Texas USA; ^3^ Department of Pediatrics McGovern Medical School at UT Health Houston, and Children's Memorial Hermann Hospital Houston Texas USA

**Keywords:** immunocompromised, malignancy, pediatric, *Stenotrophomonas maltophilia*

## Abstract

**Background:**

*Stenotrophomonas maltophilia* is a bacterial pathogen that can be fatal in hospitalized and immunocompromised patients with mortality as high as 69%. Pediatric cancer patients often have risk factors that are common for this infection, making them particularly susceptible. Managing *S. maltophilia* is especially challenging as it has inherent resistance to several antibiotics. Furthermore, soft tissue infections in neutropenic patients may deviate from the typical clinical presentation of *S. maltophilia*.

**Case Details:**

This case series describes an in‐depth examination of three cases involving immunocompromised pediatric patients with *S. maltophilia* infections. Each case exhibited a distinct clinical presentation, encompassing infection of the blood, lung, and skin, which highlights the variability in which *S. maltophilia* manifests in immunocompromised pediatric patients. These patients were treated at MD Anderson Cancer Center (MDACC) from 2020 to 2023, unfortunately resulting in fatality.

**Conclusions:**

The study aims to provide valuable insights and guidance for the management of patients with *S*. *maltophilia* infections. Emphasizing a heightened clinical suspicion will potentially lead to early initiation of directed therapy against *S. maltophilia*. Timely intervention may play a pivotal role in improving patient outcomes and reduce further burden to the healthcare system.

## 1 INTRODUCTION

*Stenotrophomonas maltophilia* is a nosocomial and opportunistic bacterial pathogen that can be fatal in hospitalized and immunocompromised patients. Although there have been many reports on *S. maltophilia* in adult immunocompromised patients, literature in pediatric cancer patients^1^ has been extremely limited, particularly necrotizing soft tissue infections.^2^, ^3^ The paucity of scientific literature regarding the management of *S. maltophilia* infections in pediatric cancer patients may result in adverse consequences, including limited awareness, incorrect therapeutic approaches, and poor patient outcomes. The aim of this case series is to raise awareness among providers of pediatric cancer patients regarding the severity of *S. maltophilia* and the course of this infection. We hope that an appropriate high index of suspicion will lead to early initiation of directed therapy and may improve outcomes.

### 1.1 Case #1

Our first patient is a 14‐year‐old female with history of mixed T cell/myeloid leukemia, refractory to multiple treatment regimens, with no previous major infectious complications. Her course was complicated by concern for hemophagolymphohistiocytosis (HLH). She was treated inpatient for HLH for 4 days at an outside institution prior to transfer to MD Anderson Cancer Center (MDACC).

One day prior to transfer to MDACC, the patient reported difficulty swallowing and was noted to have stridor. Upon arrival, she was profoundly pancytopenic. She was started on meropenem and vancomycin. Laryngoscopy showed severe swelling of the upper airway structures. She was treated with racemic epinephrine and hydrocortisone, but ultimately required emergent tracheostomy.

**FIGURE 1:**
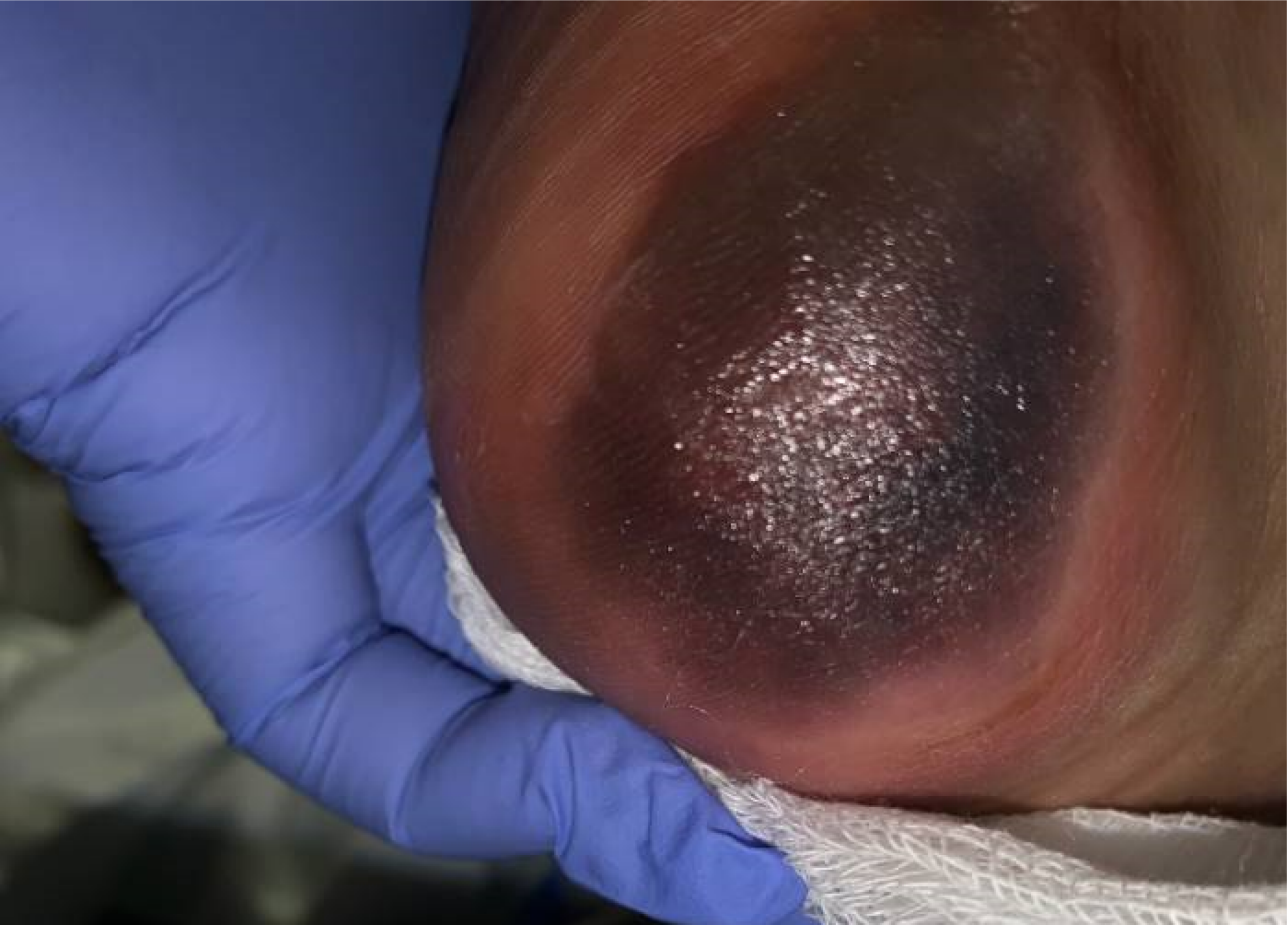
Right heel wound on day 10 of hospitalization. Note the areas of necrotic tissue at the base of the heel surrounded erythematous edema and well circumscribed.

Computerized tomography of the neck demonstrated diffuse edema throughout the submucosa of the pharynx and supraglottic larynx. Amphotericin was added with concern of invasive fungal infection. Three days after admission, her lower respiratory culture obtained during emergency tracheostomy grew moderate *S. maltophilia* susceptible to ceftazidime, levofloxacin, minocycline, and trimethoprim/sulfamethoxazole (TMP/SMX) for which she received treatment with TMP/SMX. Over the next 10 days, she had nine positive blood cultures, of which six were obtained from her central venous catheter (CVC). On day 10 of hospitalization, she also had a positive wound culture for *S. maltophilia* obtained from her right heel (Figure 1), prompting the addition of ceftazidime and minocycline and removal of CVC.

During her intensive care unit (ICU) course, she developed necrotizing pneumonia and subsequent massive, uncontrollable pulmonary hemorrhage. The patient developed septic shock secondary to *S. maltophilia* bacteremia with end organ damage requiring vasopressors and hemodialysis. Despite all interventions, the patient's clinical status continued to worsen, and she succumbed to death 12 days after admission.

### 1.2 Case #2

Our second patient was a 3‐year‐old male with acute myeloid leukemia (AML). His treatment included chemotherapy, cranial radiation and two hematopoietic stem cell transplants (HSCT). Two months after his second HSCT, he relapsed and was given venetoclax, azacitidine, and a targeted therapy under clinical trial. Since starting this treatment regimen, our patient remained pancytopenic but achieved remission. His infectious disease (ID) history was significant for *Klebsiella pneumoniae* bacteremia treated with a 14‐day course of meropenem and right leg cellulitis treated with a 7‐day course of vancomycin and piperacillin/tazobactam. He was on pentamidine prophylaxis for *Pneumocystis jiroveci* and was not on TMP/SMX prophylaxis during this time.

Our patient presented to the hospital with fever and redness on his right thigh about 5 weeks after *Klebsiella* infection. Cefepime and vancomycin were started for suspected cellulitis in the setting of febrile neutropenia. He was also given foscarnet for CMV reactivation. Pediatric surgery and ID were consulted.

Two days after admission, the patient was transferred to the ICU for hypotension and increased respiratory requirement. Cefepime was escalated to meropenem and amikacin. CT of the right lower extremity demonstrated diffuse soft tissue edema involving skin, subcutaneous fat, and intermuscular fascia, but no soft tissue gas. A wound culture the next day revealed a few coagulase negative *Staphylococcus*, although gram stain showed no white blood cells. After 2 days in the ICU, he was intubated due to hemodynamic instability in the setting of septic shock, later found to be secondary to *S. maltophilia* infection. Daily blood cultures from admission until this point remained negative. Despite aggressive measures, the patient continued to decline, parents elected to withdraw vasopressors and the patient passed away.

**FIGURE 2:**
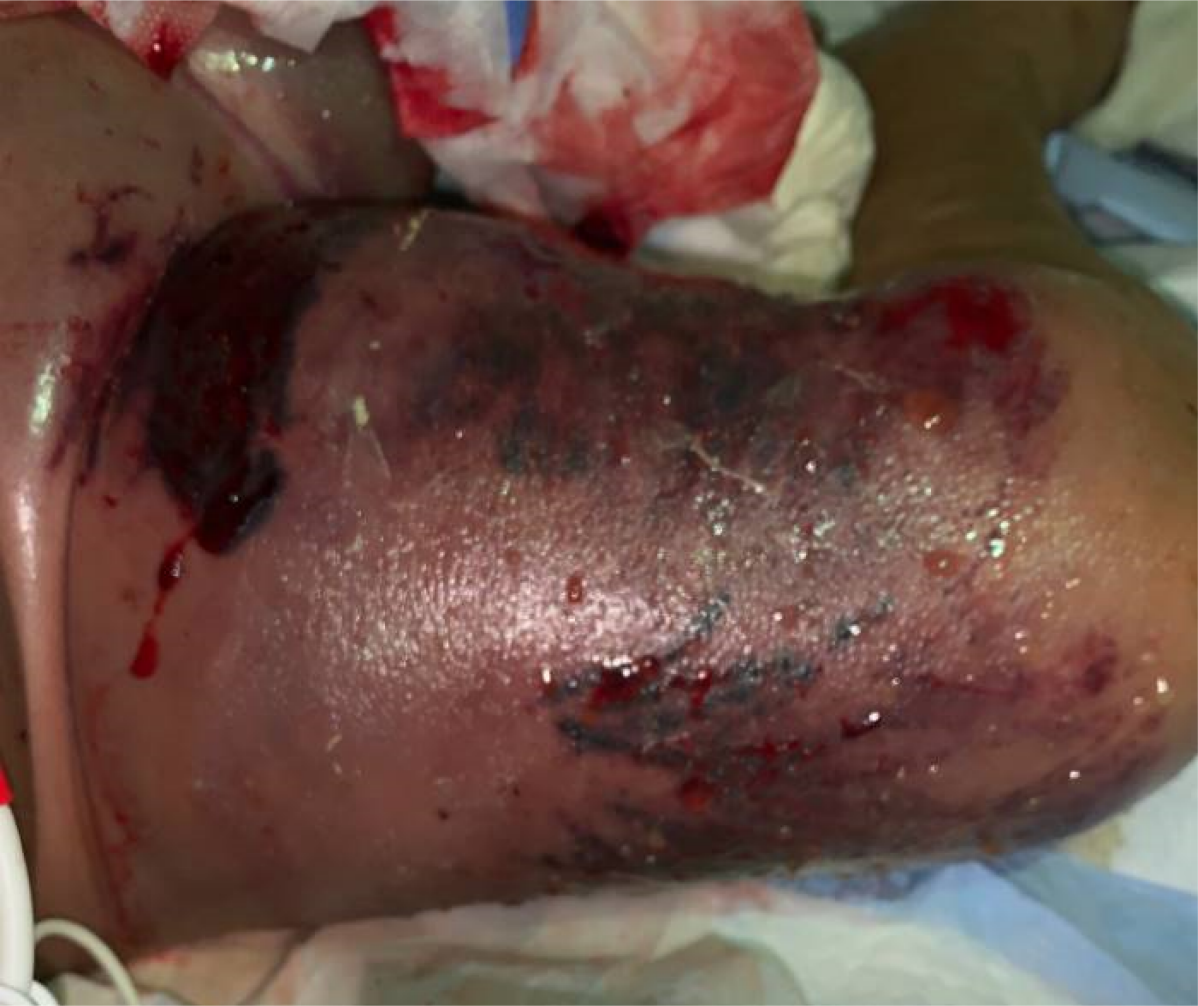
Right lower extremity shown on day 2 of hospitalization and just over 1‐year post‐HSCT. Note the areas of necrotic tissue that have evolved into open wounds with sanguineous drainage. The right lower extremity is overall very edematous and erythematous.

A superficial wound culture obtained from the right thigh on the day of demise demonstrated moderate *S. maltophilia* (Figure 2). This culture resulted after our patient passed away, and he did not receive directed antibiotics. This species was resistant to ceftazidime and levofloxacin, but susceptible to minocycline and TMP/SMX. Since our patient was on meropenem, it raises the question if this organism was a colonized specimen. Given the severity of symptoms and the timing of the wound culture, we believe this to be a true infection secondary to *S. maltophilia*.

### 1.3 Case #3

Our third patient is a 15‐year‐old male with AML, treated with chemotherapy and HSCT with myeloablative conditioning. Eight months later, he relapsed and despite starting a clinical trial targeted therapy, he demonstrated continued disease progression and restarted chemotherapy. He had a prolonged hospital course over 6 months with significant ID events in the setting of pancytopenia. He was treated for pulmonary *Aspergillus* infection with amphotericin and caspofungin. He was treated for *Staphylococcus epidermidis* bacteremia with vancomycin and daptomycin without CVC removal secondary to his critical state. He was also diagnosed with rotavirus requiring ICU care.

One month into his admission, and just over a year post transplant, his blood culture grew *S. maltophilia* and he was treated with TMP/SMX. His subsequent 28 daily blood cultures were negative for *S. maltophilia*. Two months into his admission, he developed hemoptysis concerning for pulmonary hemorrhage and possible disseminated intravascular coagulation and required transfer to ICU. There, he re‐demonstrated positive blood cultures for multidrug resistant (MDR) *S. maltophilia* and for *Mycobacterium absesscus*, both of which were treated with tigecycline. He received meropenem for 8 days, vancomycin for 7 days, amikacin for 6 days, and tigecycline for 4 days. Despite all medical interventions, he developed worsening respiratory distress and the family elected not to escalate respiratory care, and our patient passed 4 days after transfer to the ICU and about 14 months post‐transplant. His cause of death was likely secondary to multiply relapsed AML in the setting of multiple infections including *S. maltophilia*.

## 2 DISCUSSION

Our patients illustrate diverse clinical presentations of *S. maltophilia* within the oncology population, encompassing bloodstream infection, skin infection and pulmonary infection, including the occurrence of hemorrhagic pneumonia. Additionally, our cases feature less typical manifestations, including severe airway infection and soft tissue infection. All our patients were pancytopenic and severely immunosuppressed, all with refractory or relapsed leukemias. Furthermore, two of the three patients had received HSCT and the third was post‐treatment for HLH. Unfortunately, all three of our cases resulted in fatality.

Our observations from the described cases hold particular significant in the broader context of *S. maltophilia* infections. There have been very few cases of necrotizing skin and soft tissue infections, similar to our first two patients, documented in the literature.^3^, ^4^ Curiously, the first patient in our study shared the same mixed phenotype leukemia as the first adult immunocompromised patient to succumb to *S. maltophilia* necrotizing fasciitis in the Western hemisphere reported in 2018.^5^ Furthermore, the occurrence of hemorrhagic pneumonia, as seen in our first patient, has previously been described in the literature to be associated with *S. maltophilia* infections in the immunocompromised host and has been associated with high mortality in adult SCT patients.^6^

*S. maltophilia* is a gram‐negative, non‐fermenting, obligate aerobe motile bacillus.^7^ It is able to survive in nutrient poor conditions, has the ability to produce biofilm and can affect almost any organ system.^8^ Sources of infection in the hospital setting include tap water, hemodialysate, contaminated endoscopes, ventilator tubing, and CVC. *S. maltophilia* is intrinsically resistant to several antibiotics, including cephalosporins and meropenem, which are common empiric treatments for febrile neutropenia. It is usually susceptible to TMP/SMX and traditionally considered the treatment of choice (Table 1), but resistance has also been reported.^9^ Other choices for treatment include fluoroquinolones, ceftazidime, tetracyclines including tigecycline and minocycline (Table 1) while newer generation beta‐lactams like cefiderocol are reserved for MDR organisms.^10^ However, cefiderocol is only FDA indicated for patients 18 years and older.^11^ A recent study reported a 5.9% MDR resistance to TMP/SMX, 14.3% resistance to levofloxacin, and 31% resistance to ceftazidime in *S. maltophilia* bloodstream isolates from pediatric patients.^12^ Use of combination therapy has been suggested for treatment of moderate to severe infection. For instance, a study reported a combination of ciprofloxacin, trimethoprim‐sulfamethoxazole, and minocycline resulted in the longest survival time in critically ill children (*p* < 0.01).^13^ It is reasonable to consider combination therapy with fluroquinolones and tigecycline until culture sensitivities result.^3^

**TABLE 1:** Directed antibiotics for the treatment of *S. maltophilia.*

Drug	General pediatric dosing
Trimethoprim/sulfamethoxazole	8–12 mg TMP/kg/day in divided doses every 12 h (maximum dose: 160 mg TMP/dose)^14^
Levofloxacin	6 months to <5 years: 8–10 mg/kg/dose twice daily ≥5 years: 10 mg/kg/dose once daily (maximum dose: 750 mg/day)^15^
Ceftazidime	90–150 mg/kg/day in divided doses every 8 h (maximum dose: 6 g/day)^16^
Tigecycline	1–2 mg/kg/dose every 12 h (maximum dose: 50 mg/dose)^17^
Minocycline	Initial dose: 4 mg/kg once (maximum dose: 200 mg) Subsequent doses: 2 mg/kg every 12 h (maximum dose: 100 mg) (maximum daily dose: 400 mg/day)^18^

*Note*: While trimethoprim/sulfamethoxazole is usually considered the drug of choice for *S. maltophilia*, final treatment decisions will depend on susceptibility testing.

While not usually considered a virulent pathogen, *S. maltophilia* can be devastating in an immunocompromised patient. Mortality for *S. maltophilia* has been reported as high as 69%^19^ but varies depending on risk factors. A systematic review of 15 articles concluded an attributable mortality rate of up to 37.5% in patients with *S. maltophilia* infection.^20^ A retrospective review of 142 adult patients with bacteremia reported a 36.6% mortality within 28 days of diagnosis.^19^ A retrospective study of 68 pediatric patients reported a 33.8% mortality within 7 days of *S. maltophilia* bacteremia.^12^ A multicenter retrospective review of 68 pediatric bacteremic patients reported 42% mortality.^13^ For post HSCT patients, a retrospective review reported mortality from any organ system in 10 pediatric patients at 100 days as 50–60%.^3^ A multicenter retrospective review of 11 HSCT patients compared to 68 pediatric cancer patients with *S. maltophilia* in any organ revealed survival rates significantly lower in HSCT than in pediatric oncology patients (45% vs. 85%, *p* = 0.001).^21^ Adult studies have shown lack of removal of CVC, sepsis upon presentation and infection prior to engraftment in the setting of transplant to be associated with increased mortality.^6^

The purpose of this case series is to review our experience *with S. maltophilia* and to raise awareness for this fatal pathogen in immunocompromised pediatric patient population, and pediatric HSCT population. Severe pulmonary illness and soft tissue infections in patients with history of broad and/or prolonged antibiotic exposure, especially if not responsive to initial empiric antibiotic therapy, should raise concern for *S. maltophilia* infection and prompt empiric treatment to reduce mortality with guidance from ID consultants. Combination therapy may have to be considered for proven infections in this patient population until antibiotics sensitivities have resulted and the patient is not critically ill.^3^, ^22^ It is necessary to remove affected central lines if the patient is stable and removal is safe, or surgically debride infected areas early on if possible, with guidance from surgeons. In addition, it is very important to be mindful of antibiotic stewardship and de‐escalating broad spectrum antibiotics such as meropenem when there is no organism identified to treat. As this *S. maltophilia* is a nosocomial pathogen that can be devasting in the immunocompromised patient population, it is very important to also involve hospital infection control. Consequences of inadequate infection control could result in an outbreak of *S. maltophilia* cases with a high rate of mortality. Given the rare yet devastating nature of *S. maltophilia* invasive infection, our team is proactively responding to our cases by incorporating practice changes to our clinic. Specifically, we have established a comprehensive assessment of *S. maltophilia* history in standardized patient handoffs. In addition, we are in the process of implementing a research initiative to collect further data to better understand the prevalence and management of *S. maltophilia* in pediatric cancer patients. Since the enactment of these measures, we have not observed any further cases of *S. maltophilia* within our department. To our knowledge, these three cases have been the only cases of *S. maltophilia* at our institution from 2020 to 2023, and had 100% mortality.

In conclusion, our case series highlights the importance of early recognition and tailored management of *S. maltophilia* infections in pediatric cancer patients. Clinical insights gained from this study underscore the importance of collaboration with ID specialists and the role of infection control practices to mitigate the consequences of *S. maltophilia* infections in immunocompromised patient populations.

## AUTHOR CONTRIBUTIONS

**Sanila Sarkar:** Conceptualization (lead); writing – original draft (lead); writing – review and editing (lead). **Lea M. Stitzlein:** Writing – review and editing (supporting). **Emily Rav:** Writing – review and editing (supporting). **Miriam B. Garcia:** Writing – review and editing (supporting). **Michael B. Chang:** Conceptualization (supporting); writing – original draft (supporting). **Ramia Zakhour:** Writing – original draft (supporting); writing – review and editing (supporting).

## CONFLICT OF INTEREST STATEMENT

The authors have stated explicity that there are no conflicts of interest in connection with this article.

## ETHICS STATEMENT

Per University of Texas MD Anderson Cancer Center Institutional Review Board, this project is defined as a case series and has therefore received waiver of informed consent and authorization by the per institutional policy. All patient information has been deidentified.

## Data Availability

No data was analyzed in this manuscript.
